# Basal Cell Carcinoma in Perianal Region: A Rare Case Presentation and Review of Literatures

**DOI:** 10.7759/cureus.101626

**Published:** 2026-01-15

**Authors:** Sudhir Singh, Manish Kumar, Shashi S Pawar, Anushweta Singh, Prabhjot Ahluwalia

**Affiliations:** 1 Surgical Oncology, Indira Gandhi Institute of Medical Sciences, Patna, IND; 2 Pathology, Indira Gandhi Institute of Medical Sciences, Patna, IND

**Keywords:** basal cell carcinoma, human papillomavirus, immunosuppression, perianal, ultraviolet radiation

## Abstract

Basal cell carcinoma (BCC) is the most common form of non-melanoma skin cancer, typically arising in areas frequently exposed to ultraviolet (UV) radiation, such as the face and neck. Occurrence in sun-protected areas is uncommon, and perianal or genital involvement is particularly rare. In this report, we present the case of a 64-year-old patient diagnosed with perianal BCC, who lacked the commonly associated risk factors like UV radiation exposure and immunosuppression. This case highlights the need for clinical awareness of atypical presentations of BCC. Patient underwent wide local excision of the lesion with clear margins. Anal sphincter was preserved without compromising the margin status. The surgical defect was reconstructed with an advancement flap. Postoperative recovery was uneventful. Histopathological examination confirmed the diagnosis of nodular BCC with the closest margin 5 mm, which was the medial margin. Immunohistochemistry was positive for Ber-EP4 and BCL2. The patient has been kept on a three-month follow-up. History and physical examinations were performed on every follow-up. Patient is recurrence-free till the last follow-up.

## Introduction

Basal cell carcinoma (BCC) is a frequently encountered cutaneous malignancy, accounting for approximately 75% of nonmelanocytic skin tumors [1]. Over 80% of BCC cases develop in sun-exposed regions of the body, particularly the head and neck. Ultraviolet (UV) radiation is considered the primary etiological factor in BCC pathogenesis. Additional risk factors include increasing age, male sex, immunosuppression, and exposure to carcinogens such as arsenic. BCC may also arise in areas of chronic inflammation, burn scars, ulcers, or pre-existing squamous cell carcinomas [1,2]. Apart from increasing age and male gender, no common risk factors, such as UV radiation exposure and immunosuppression, were associated with our case. However, BCC occurrence in UV-protected areas, especially in the genital and perianal regions, is exceedingly rare and may pose a diagnostic challenge. Incidence of basal cell carcinoma is rising, and hence, there is a need for preventive measures.

Squamous cell carcinoma (SCC) is the most common type of anal cancer. Perianal BCC is the rarest among all anorectal malignancies. However, basaloid SCC, which shares overlapping histological features with BCC, found in the anal canal, has a higher risk of distant metastasis compared to BCC [3]. The prognosis for basaloid carcinoma is worse than that for BCC. Perianal BCC tends to be a regional disease. SCC is treated with definitive concurrent chemoradiation therapy, and BCC is typically treated with local excision with negative margins. It is important to differentiate between these two types of cancer, as there is a difference in treatment modality.

Various treatment modalities can be used for treating perianal BCC, including wide local excision, Mohs microscopic surgery, or radiotherapy. While topical imiquimod has been shown to be effective in treating superficial BCCs, its role in the treatment of perianal BCC is not proven. A standard excision with negative margin is recommended treatment for BCC, while radiotherapy is used for patients who are unable to undergo surgery. Systemic treatments, such as inhibitors of the Hedgehog signaling pathway, are recommended for treating patients with recurrences at distant sites and unresectable local recurrences [4]. Vismodegib and Sonidegib are two approved Hedgehog signaling pathway inhibitors for locally advanced and metastatic BCC.

Perianal BCC can resemble benign conditions, such as hemorrhoids, fistulas, fissures, or infections, making it crucial for surgeons to consider the possibility of malignancy before surgery.

## Case presentation

**Figure 1:**
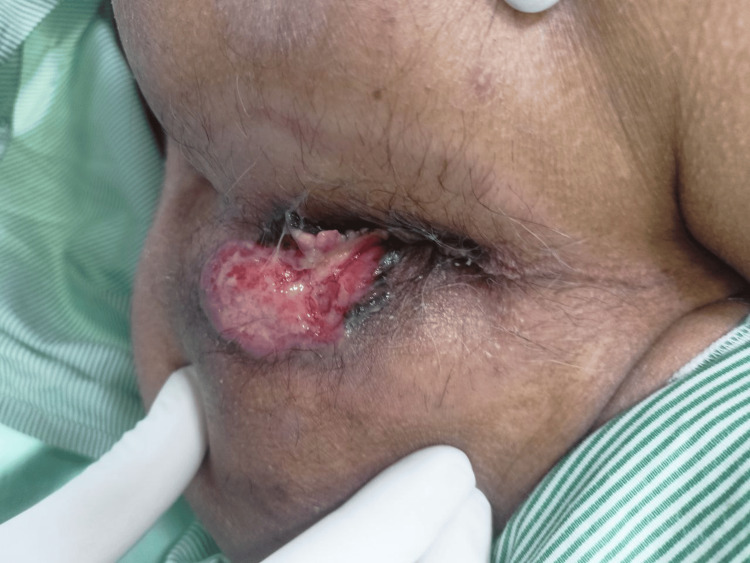
Pre-operative photograph of BCC in the left perianal region BCC: Basal cell carcinoma

**Figure 2:**
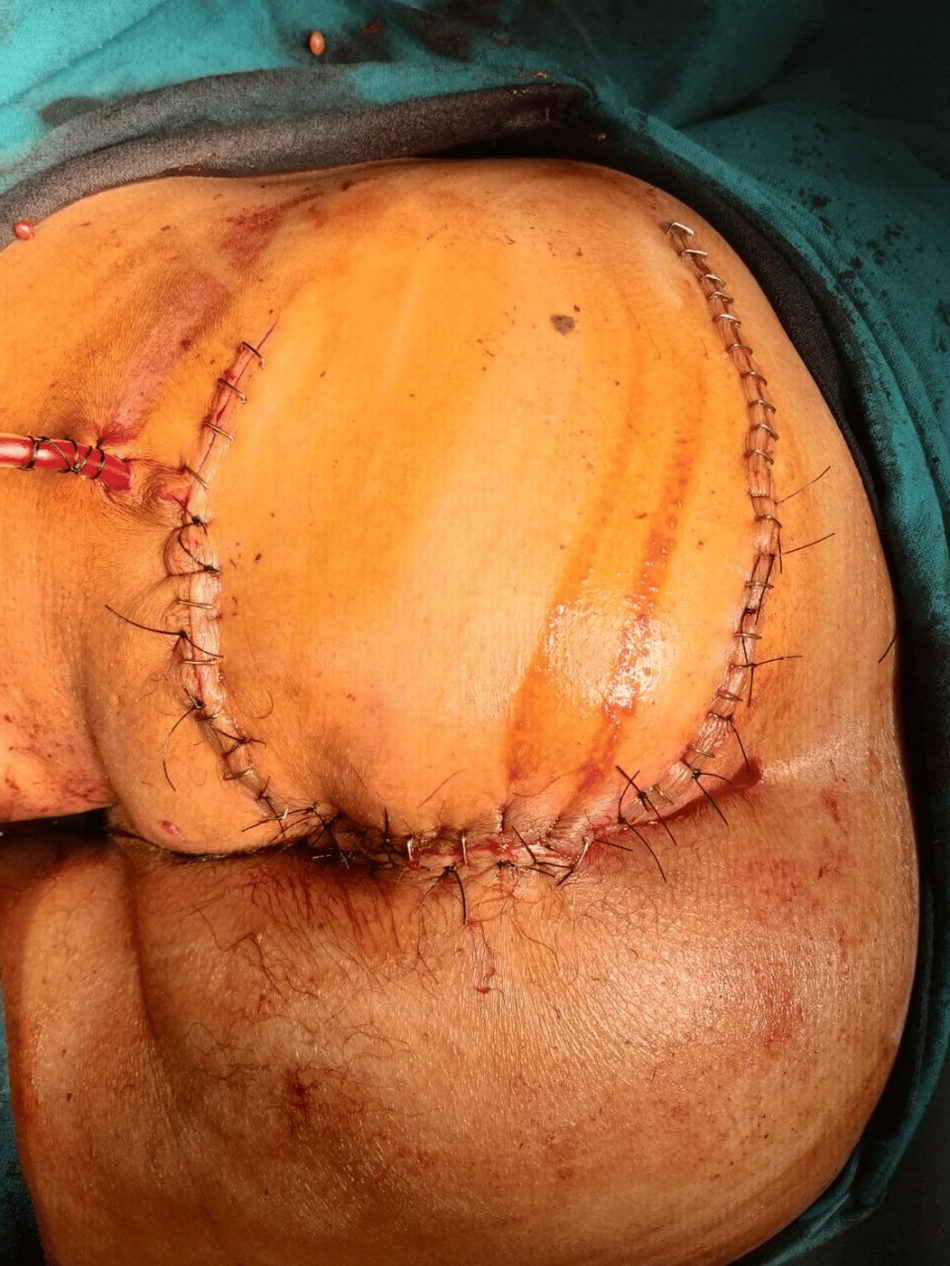
Postoperative photograph showing coverage of the defect with an advancement flap

**Figure 3:**
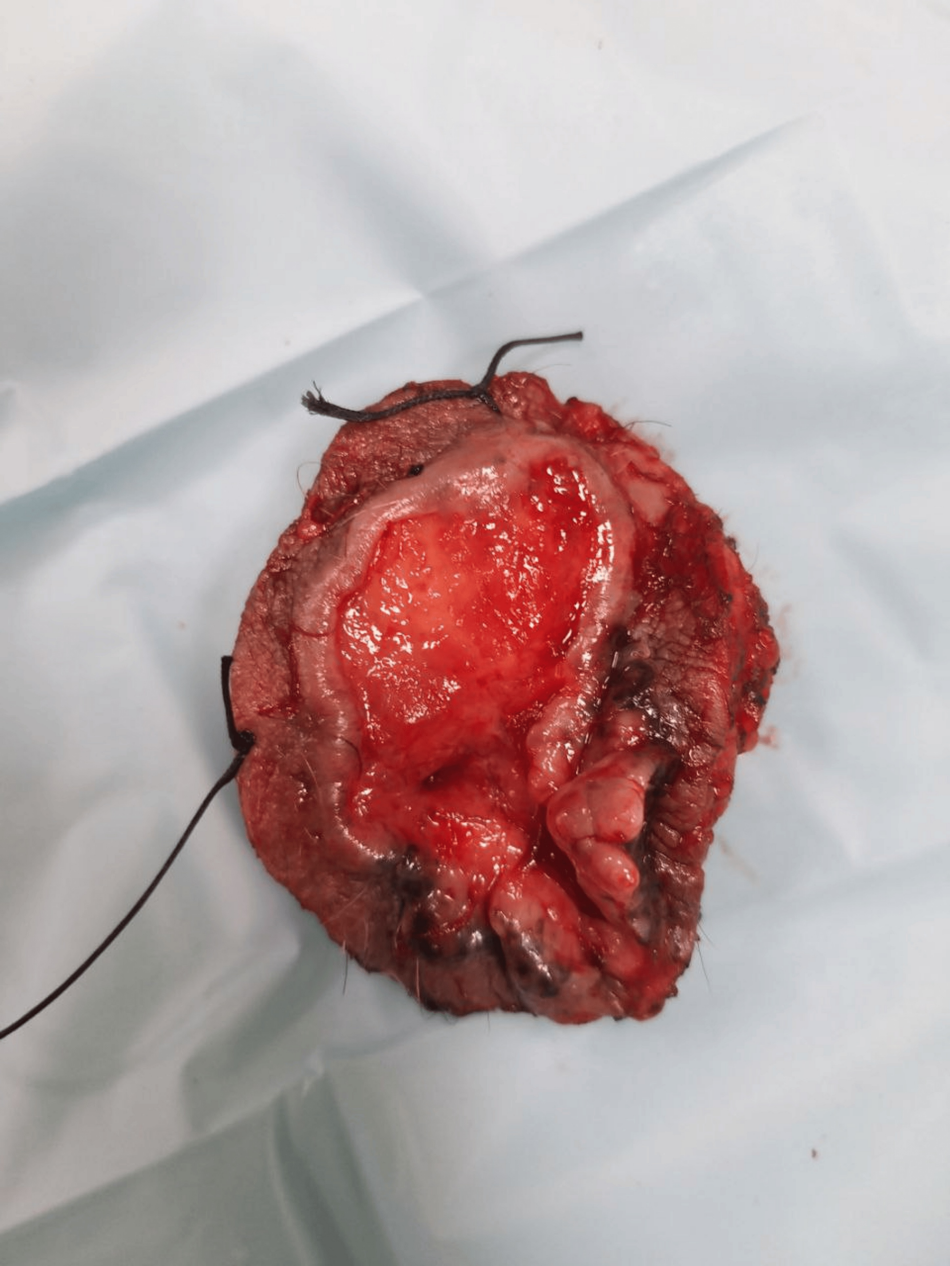
Excised specimen of BCC left perianal region Short suture denotes the superior margin and long suture denotes the lateral margin. The lesion measures 5×4 cm. BCC: Basal cell carcinoma

**Figure 4:**
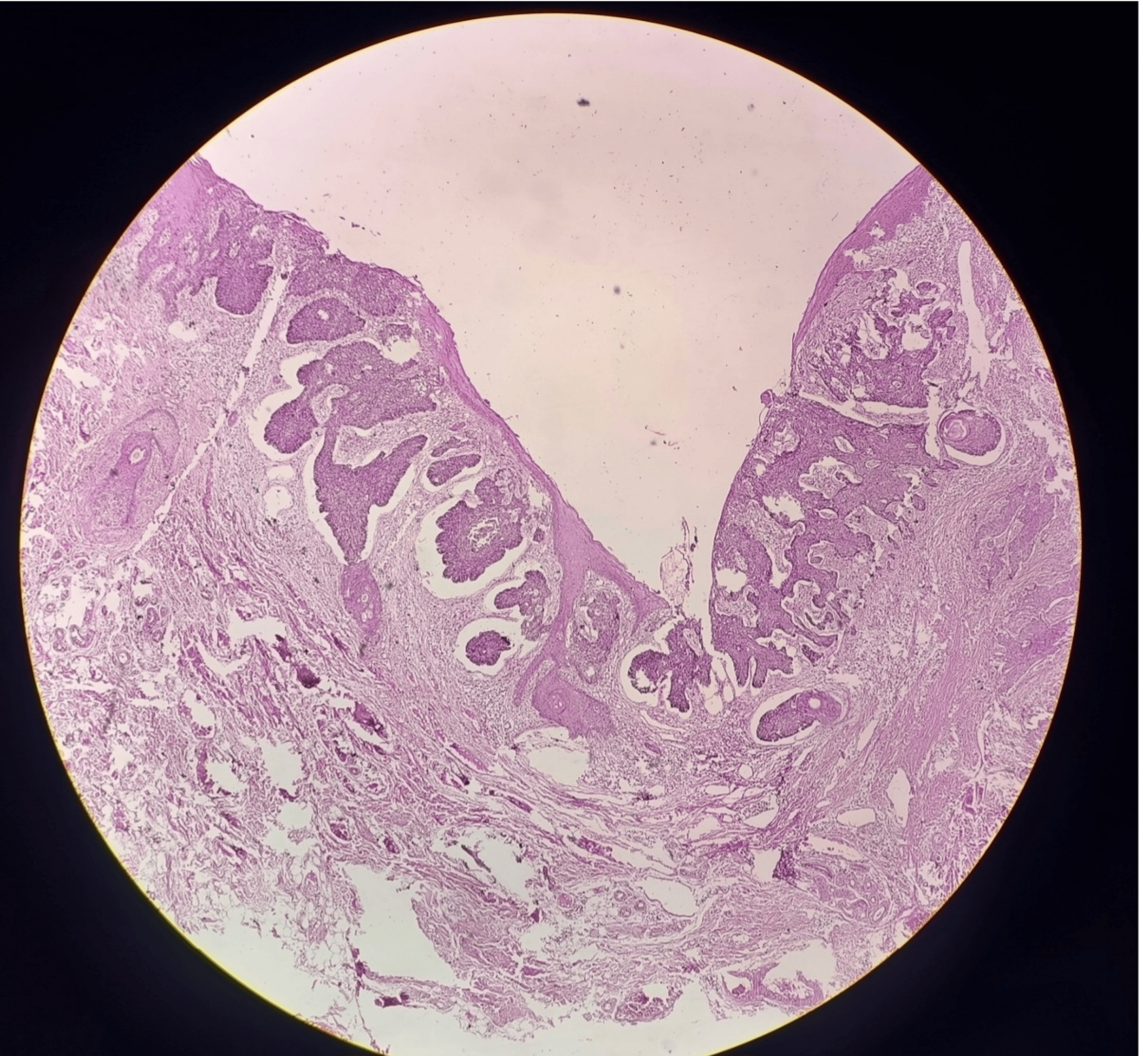
Nodular BCC- large basaloid lobules with peripheral palisading and epidermal attachment (H&E ×40) BCC: Basal cell carcinoma

**Figure 5:**
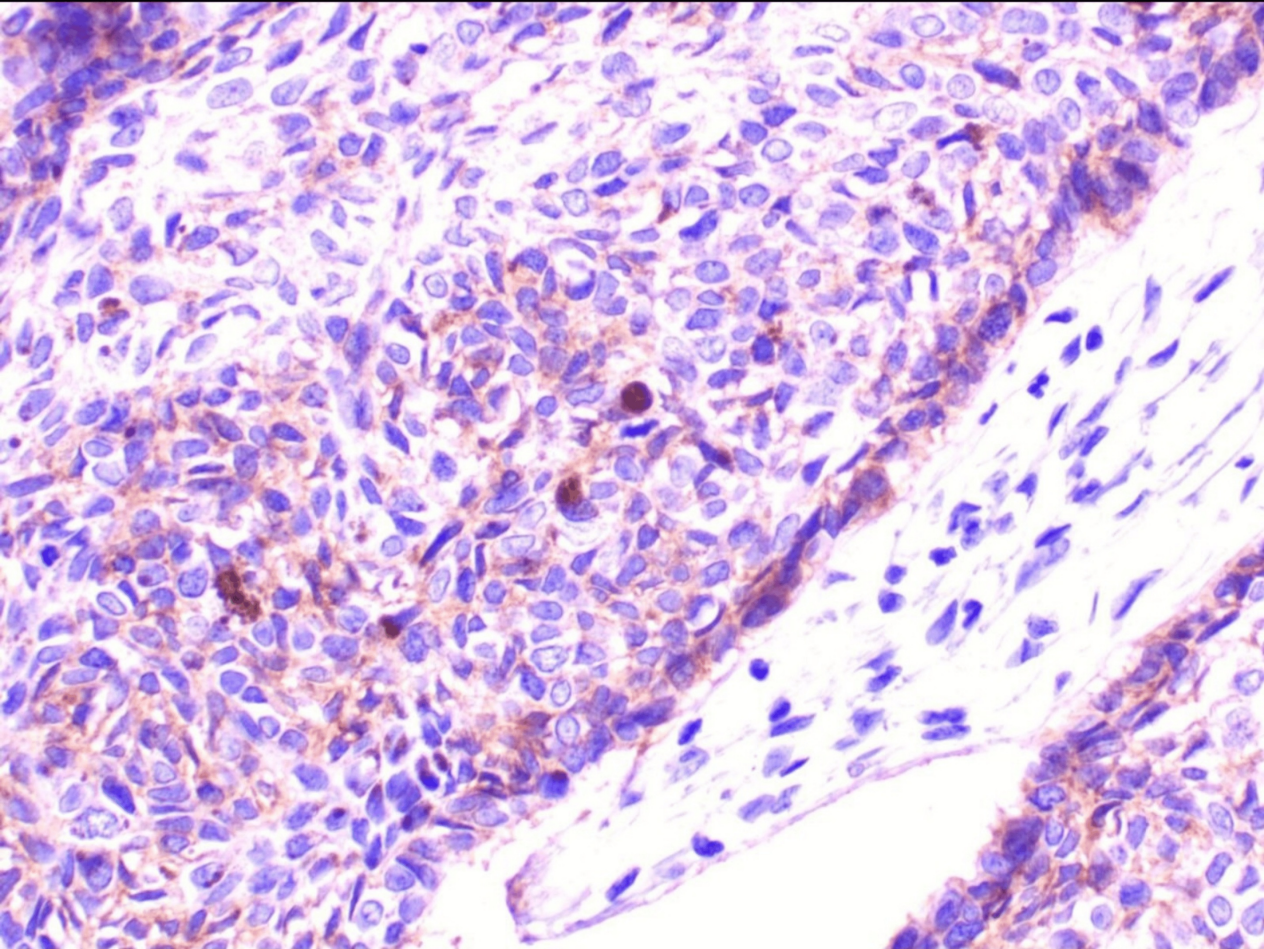
Immunohistochemical staining showing focal positivity for Ber EP4

**Figure 6:**
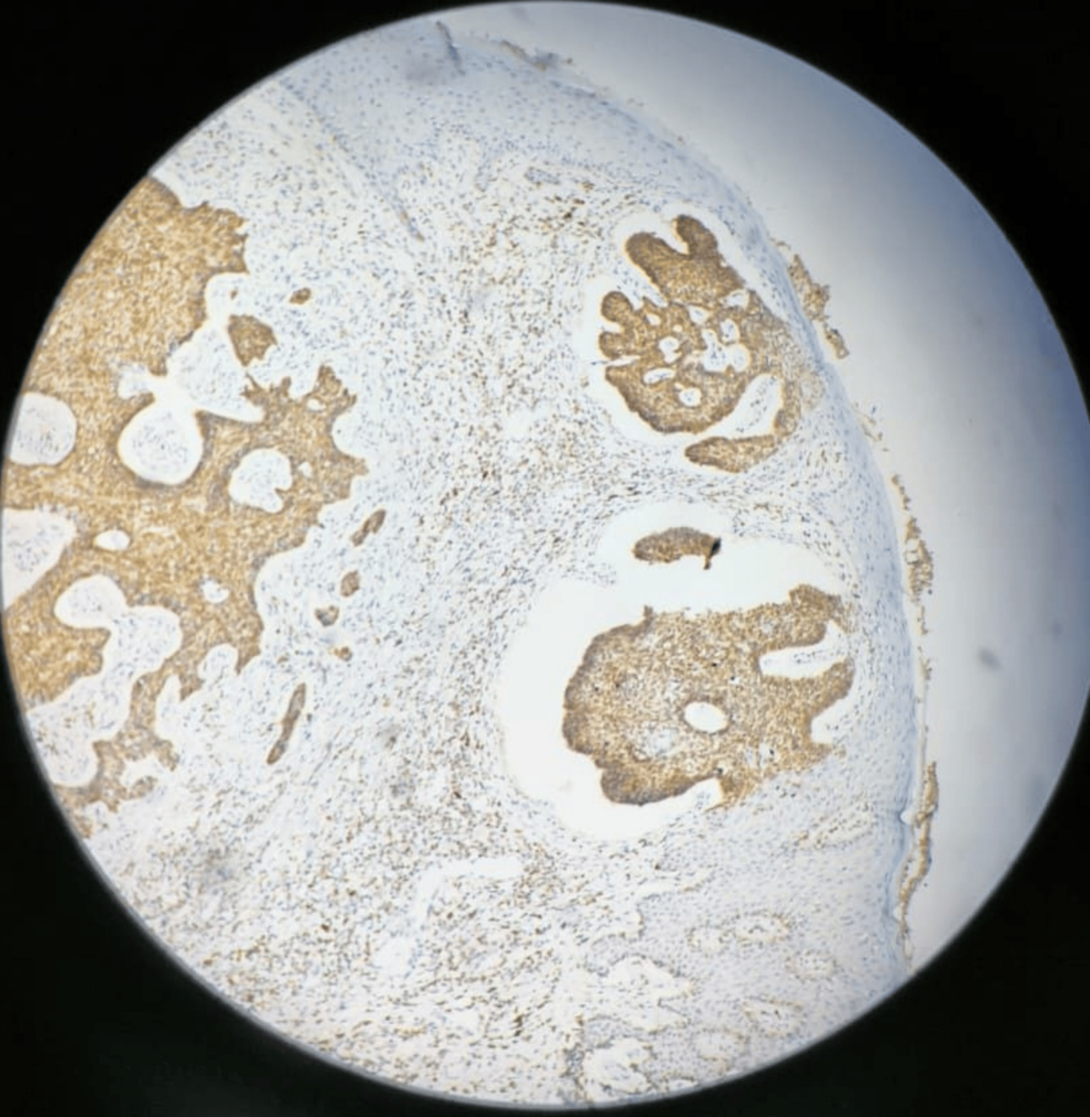
Immunohistochemical staining showing diffuse positivity for BCL2

A 64-year-old gentleman presented with a left perianal ulcerated lesion of size 5×4 cm for the last 1 year (Figure 1). It was gradually progressive, painless, and associated with serosanguinous discharge. There was no history of radiation therapy to the local site. There was no history of chronic wound or sinus at the lesion site. There was no history of immunosuppression. There was no history of Icterus, abdominal lump, or cough. The patient was a known hypertensive on medication. There was no history of prior medical and surgical treatment for the lesion. On examination, the lesion was in the left perianal region till the anal verge without sphincter involvement. There was black pigmentation at the medial edge of the lesion. The lesion had rolled out edges and bled to the touch. The lesion was not fixed to the underlying muscle. On abdominal examination, there was no palpable lump or organomegaly. Bilateral inguinal lymph nodes were not palpable. Per Rectal examination was unremarkable. Contrast-enhanced magnetic resonance imaging (CEMRI) showed a 4 × 2 cm lesion in the left perianal region without anal sphincter involvement. The HPV test was negative. A wedge biopsy was performed from the lateral edge of the lesion, incorporating subcutaneous fat under local anesthesia. Histopathological examination of the biopsy showed features of nodular BCC. Patient underwent wide local excision of the lesion with gross circumferential 1 cm normal margin, including subcutaneous fat till deep fascia. Anal sphincter was preserved. The surgical defect was reconstructed with an advancement flap (Figures 2-3). Postoperative recovery was uneventful. Final histopathological examination of the excised specimen showed features of nodular BCC (Figure 4). All margins were free from tumor cells. The closest margin was the medial margin, which was 5 mm. Immunohistochemical staining was positive for Ber EP4 and BCL2, supporting the diagnosis of BCC (Figures 5-6). Patient has been kept on a 3-month follow-up with history and physical examination and is recurrence-free for one year.

## Discussion

Perianal BCC is an exceptionally rare presentation, accounting for approximately 0.2% of all anorectal cancers and only about 1% of all BCC cases [5,6]. Although BCC commonly arises in sun-exposed areas, its occurrence in the perianal region highlights the importance of recognizing alternative pathogenic mechanisms beyond ultraviolet (UV) radiation.

Apart from increasing age and male gender, there were no risk factors present in our case, such as UV radiation exposure, immunosuppression, and chronic sinus.

The literature indicates that perianal BCC is more prevalent in males, with a reported mean age of 73 years at diagnosis [5]. Several contributing factors have been proposed in the etiopathogenesis of perianal BCC, including chronic irritation or trauma (such as persistent pruritus or dermatitis), nevoid BCC syndrome, mutations in the tumor suppressor gene p53, immunodeficiency states, sexually transmitted infections, and exposure to arsenic [5,7]. Unlike SCCs in the anogenital region, perianal BCC has not been linked to human papillomavirus (HPV) infection, suggesting a distinct pathogenesis [8].

The differential diagnosis for perianal BCC is broad and includes conditions such as condyloma acuminatum, Bowen’s disease, extramammary Paget’s disease, and SCC. It is especially important to differentiate perianal BCC from basaloid (cloacogenic) squamous cell carcinoma, which is known for its aggressive behavior and early metastatic potential [8]. Histopathological and immunohistochemical evaluation play a crucial role in distinguishing between these entities. The classic histological features of BCC include peripheral palisading of tumor cell nuclei and stromal retraction. Immunohistochemically, strong expression of Ber-EP4 and BCL2 supports the diagnosis of BCC [8,9]. Patil et al. noted that the absence of tumor retraction artifact and atypical mitotic figures, along with the presence of strong Ber-EP4 and BCL2 staining, are indicative of BCC rather than other basaloid neoplasms [3]. In our case, histopathological examination and immunohistochemistry for BCL2 and Ber-EP4 were used for establishing the diagnosis of BCC.

HPV-associated markers, such as p16(INK4a) overexpression, are commonly present in anal squamous cell carcinomas but have not been implicated in perianal BCC, further supporting the distinct etiopathological profile of this tumor type [10,11].

In our case, the diagnosis of perianal BCC was confirmed based on histopathological findings consistent with the nodular subtype, which is the most common histological variant in this anatomical location [3,5]. Gibson and Ahmed reported that the superficial subtype is the second most common, accounting for approximately 18% of perianal and genital BCCs [5]. Similarly, Patil et al. identified one nodular/superficial and one superficial BCC case among nine perianal lesions evaluated histologically [3].

This case shows the importance of including BCC in the differential diagnosis of perianal lesions, even in the absence of traditional risk factors such as UV exposure or immunosuppression. Careful examination and histopathological confirmation are essential for accurate diagnosis and appropriate management. Perianal lesions should undergo pre-operative biopsy to confirm the diagnosis so that an appropriate treatment modality can be used for the specific diagnosis.

## Conclusions

Although BCC is typically associated with sun-exposed areas, rare presentations in UV-protected regions, such as the perianal area, should be considered in the differential diagnosis of chronic, non-healing perianal lesions. Histopathological evaluation, supported by immunohistochemical staining, is necessary for accurate diagnosis. Clinicians should maintain a high index of suspicion, particularly in elderly patients, even in the absence of conventional risk factors. Timely recognition and appropriate surgical management can result in excellent outcomes with the least risk of recurrence.

In this case, the diagnosis of BCC was confirmed by pre-operative wedge biopsy and immunohistochemistry. Wide local excision with clear margins was done. Patients need 3-6-month follow-ups initially for 5 years, then at least annually for life-long.
